# Clinical significance of HER2 overexpression in biliary tract carcinoma ——a meta analysis

**DOI:** 10.3389/fonc.2025.1534005

**Published:** 2025-05-14

**Authors:** Haonan Xu, Yuwen Liang, Wenqiang Tang, Xiongxin Yang, Xiaobo Du

**Affiliations:** ^1^ Department of Oncology, National Health Commission Key Laboratory of Nuclear Technology Medical Transformation, Mianyang Central Hospital, Mianyang, Sichuan, China; ^2^ Department of Oncology, Affiliated Hospital of North Sichuan Medical College, Nanchong, Sichuan, China; ^3^ Sichuan Clinical Research Center for Radiation and Therapy, Mianyang Central Hospital, Mianyang, Sichuan, China; ^4^ Department of Hepatobiliary Surgery, Affiliated Hospital of North Sichuan Medical College, Nanchong, Sichuan, China

**Keywords:** HER2, Biliary tract carcinoma, clinicopathological features, prognosis, Meta-analysis

## Abstract

**Introduction:**

Previous studies have been inconsistent on the correlation of human epidermal growth factor receptor 2 (HER2) overexpression in biliary tract carcinoma. The objective of this meta analysis was to assess its association with clinicopathological features and prognostic significance of biliary tract carcinoma.

**Methods:**

The eligible studies were searched in Pubmed, Embase and Web of Science databases. Inclusion criteria were studies of the relationship between HER2 positive expression (ICH: HER2 (+++), FISH: HER2 overexpression, NGS: HER2 overexpression) and prognosis or clinicopathological features of patients with biliary tract carcinoma (BTC). The analysis was conducted according to gender, high differentiation degree, middle differentiation degree, tumor stage, nerve invasion, vascular invasion, lymph node metastasis and pathological diagnosis of patients. ES (Effect Sizes) for 95% confidence intervals (CI) were calculated to examine risk or hazard associations, and heterogeneity and sensitivity analyses were performed.

**Results:**

A total of 15 studies were included to evaluate the association of HER2 positive expression with clinicopathological features and survival prognosis. There was no significant statistical relationship between positive/high expression of HER2 and a series of clinical characteristics including gender, high, middle and low differentiation, tumor stage, vascular invasion, nerve invasion, Lymph node metastasis, T stage and pathological type of patients with biliary tract carcinoma. There was a significant relationship between positive/high expression of HER2 and postoperative Disease-Free Survival in patients with biliary tract carcinoma (ES = 1.87, 95% CI: 1.24-2.81, p = 0.003). There was a significant relationship between positive/high expression of HER2 and postoperative Overall Survival in patients with biliary tract carcinoma (ES = 1.54, 95% CI: 1.08-2.20, p = 0.017).

**Discussion:**

Our meta-analyses revealed that high expression of HER2 gene was not correlated with clinicopathological parameters such as differentiation degree, TNM stage, lymph node metastasis, vascular invasion, nerve invasion, pathological type, T stage, and gender of biliary tract carcinoma. HER2 overexpression is a negative prognostic factor in biliary tract carcinoma patients. The association between positive/high expression of HER2 and the pathological features as well as prognosis in biliary tract carcinoma patients warrants further validation.

## Introduction

Biliary tract carcinoma (BTC) is a malignant tumor originating in the epithelium of the biliary tract, including Intrahepatic cholangiocarcinoma (ICC). extrahepatic cholangiocarcinoma (eCCA) and gallbladder carcinoma (GBC), Extrahepatic cholangiocarcinoma is further divided into perihilar cholangiocarcinoma (pCCA) and distal cholangiocarcinoma (dCCA). BTC accounts for 3% of all gastrointestinal malignancies (1, 2). Among them, ICC is the second most common primary liver malignancy after hepatocellular carcinoma, accounting for 10% to 15% (3). The incidence of BTC is on the rise globally and is generally higher in Asian countries than in Western countries, especially China, Thailand and South Korea (4). As a malignant tumor with a very high degree of malignancy and a very poor prognosis, the molecular biological pathogenesis and the following factors of BTC has not been fully clarified. Currently, it is believed that the following factors may be related: such as bile duct stones, cholangitis, Clonorchis sinensis infection, primary sclerosing cholangitis, chronic liver diseases (liver cirrhosis and viral infections). At least four genetic diseases can increase the risk of cholangiocarcinoma, including Lynch syndrome, BRCA-associated protein-1 (BAP1) tumor susceptibility syndrome, cystic fibrosis, and biliary papillomatosis. According to the epidemiological investigations and analyses of cholangiocarcinoma in the East and West, in Asian countries, the main risk factors for cholangiocarcinoma are bile duct stones and cholangitis. The prevalence of bile duct stones is relatively high in Asian countries. Long-term stimulation of the bile duct wall by stones, combined with chronic cholangitis, creates a high-risk environment for canceration; while in developed Western countries, the main risk factors are bile duct malformations or metabolic syndrome, such as the incidence of primary sclerosing cholangitis is much higher than in Asian regions (5–7).Early-stage cholangiocarcinoma has no specific clinical symptoms. As the disease progresses, jaundice, abdominal distension, skin itching, dark urine and pale stools may occur. Accompanying symptoms may include listlessness, weight loss, fatigue, steatorrhea and weight reduction. Due to the lack of obvious clinical manifestations in the early stage of cholangiocarcinoma, it is not until the middle and late stages when biliary obstruction causes clinical symptoms that the disease attracts attention. By this time, the best opportunity for surgical treatment has been missed, resulting in poor clinical outcomes and prognosis (8). Therefore, early diagnosis and prevention are particularly important. In recent years, exploring new diagnostic and prognostic factors has become the focus of diagnosis and treatment of cholangiocarcinoma (9). The discovery of biomarkers with high sensitivity and high specificity is of great significance for early diagnosis, early treatment, improvement of prognosis and survival rate of patients.

Human epidermal growth factor 2 (HER2) encodes a 185 kDa transmembrane tyrosine kinase, and overexpression of the HER2 gene is associated with poor prognosis in a variety of human cancers, such as breast, ovarian, and lung cancers (10). For example, HER2 overexpression can be detected in 25-35% of breast cancers (11). Previous TCGA database analysis has revealed the potential molecular mechanism of HER2 overexpression promoting tumor development, and the relevant experimental results have been verified: the ERBB signaling pathway where HER2 gene is located is closely related to cell proliferation and migration, and its amplification may drive tumor progression through activation of the downstream MAPK and PI3K-AKT pathways (12). HER2 amplification is co-expressed with mTOR pathway genes (such as RICTOR), suggesting that it supports tumor growth through metabolic reprogramming (13). These results suggest that HER2 amplification is closely related to the occurrence and development of malignant tumors, and the prognosis of malignant tumor patients with HER2 amplification may be worse, which provides potential guidance for clinical practice. For the treatment of advanced biliary tract carcinoma, chemotherapy is the cornerstone of treatment. With the continuous exploration of advanced treatment, such as clarifying the important molecular targets of tumor FGFR2 fusion/rearrangement, IDH1/2 mutation, HER amplification, MSI-H/dMMR, etc., targeting drugs or immune drugs are added on the basis of traditional chemotherapy. The survival of such patients was prolonged (14, 15). At present, a large number of studies have been published on the expression of HER2 in Biliary tract carcinoma and its relationship with clinicopathological features of biliary tract carcinoma, suggesting a certain relationship between the two. However, each study had a small sample size and produced different results. Therefore, the purpose of this study was to evaluate the correlation between HER2 overexpression in biliary tract carcinoma and clinicopathological features and prognosis of biliary tract carcinoma through meta-analysis, providing a reference for clinical assessment of biliary tract carcinoma.

## Materials and methods

### Literature search

A systematic evaluation and meta-analysis was conducted following the Preferred Reporting Items for Systematic Reviews and Meta-analyses (PRISMA) statement and the PRISMA extension statement for meta-analysis (16). The Chinese databases (Wanfang, VIP, CNKI, CBM) and English databases (PubMed, The Ochranelibrary, Web Science, Embase) were searched for all the articles related to HER2 and biliary tract carcinoma. The retrieval time was from self-established database to August 2024. Search terms include: HER2, biliary tract carcinoma(BTC), intrahepatic cholangiocarcinoma(ICC), extrahepatic cholangiocarcinoma(eCCA), gallbladder carcinoma(GBC), perihilar cholangiocarcinoma (pCCA) and distal cholangiocarcinoma (dCCA).

### Inclusion and exclusion criteria

Meet the following inclusion criteria of the study was taken into account: (a) patients with biliary pathology diagnosis of malignant tumor. (b) To discuss the relationship between HER2 and clinicopathological features and prognosis. Exclusion criteria were as follows:(a) literature not related to HER2 or biliary tract carcinoma; (b) Literature on secondary biliary tract carcinoma; (c) Similar studies by the same author and multiple duplicate data in different works; (d) Estimates of effects in animal experiments, case reports, correspondence, reviews, expert opinions, letters, talks or summaries of meetings, and studies that have not been fully published.

### Data extraction and quality assessment

Two reviewers (Liang Yuwen and Tang Wenqiang) extracted the data independently, and any disputes between the two reviewers were resolved by consensus by the third reviewer (Xu Haonan). For each included study, the following data were extracted: year of publication; Name of the first author; Country, number of patients, trial design, treatment regimen, high HER2 expression ratio, clinicopathological parameters such as sex, age, tumor size (T), lymph node metastasis (N), perineuronal invasion, vascular invasion, tumor differentiation, AJCC TNM stage, OS or DFS; Results of univariate and/or multifactorial analyses (including HR, P-value, and 95% CI).

Quality evaluation was conducted independently by three researchers (Liang Yunwen, Yang Xiongxin andTang Wenqiang). The Newcastle-Ottawa Quality Assessment Scale was used to assess the quality of the study (17). All statistical analyses were performed using STATA version 18.0.

### Statistical analysis

STATA 18.0 software was used for the meta-analysis. Odds ratio (OR) and confidence interval (ci) were used for binary categorical variables. Calculated mean difference (MD) or standardized mean difference (SMD) and 95% confidence interval (CI) for continuous outcome measures. The χ2 test was used to determine the heterogeneity among the studies. Because there is always heterogeneity between multiple studies across different populations and geographic locations, we used a random effects model for meta-analysis. The I2 statistic was used to evaluate the efficacy of all heterogeneity. In addition, we used the Chi2 heterogeneity test to determine the heterogeneity of the true strength of the evidence. According to statistical criteria, heterogeneity was classified as insignificant (I2: 0%-40%), moderate (I2: 30%-60%), significant (I2: 50%-90%), or equivalent (I2: 75%-100%) (18). If there were overlapping values, it was necessary to observe the I2 values: i) the magnitude and direction of the effect; and ii) the strength of evidence of heterogeneity (e.g., P-values obtained from χ2 tests, or CIs for I2) If the heterogeneity is too large to be resolved, a descriptive analysis is performed. Funnel plot, Begg’s test and Egger’s test were used to evaluate publication bias (19).

## Result

### Search results

**Figure 1:**
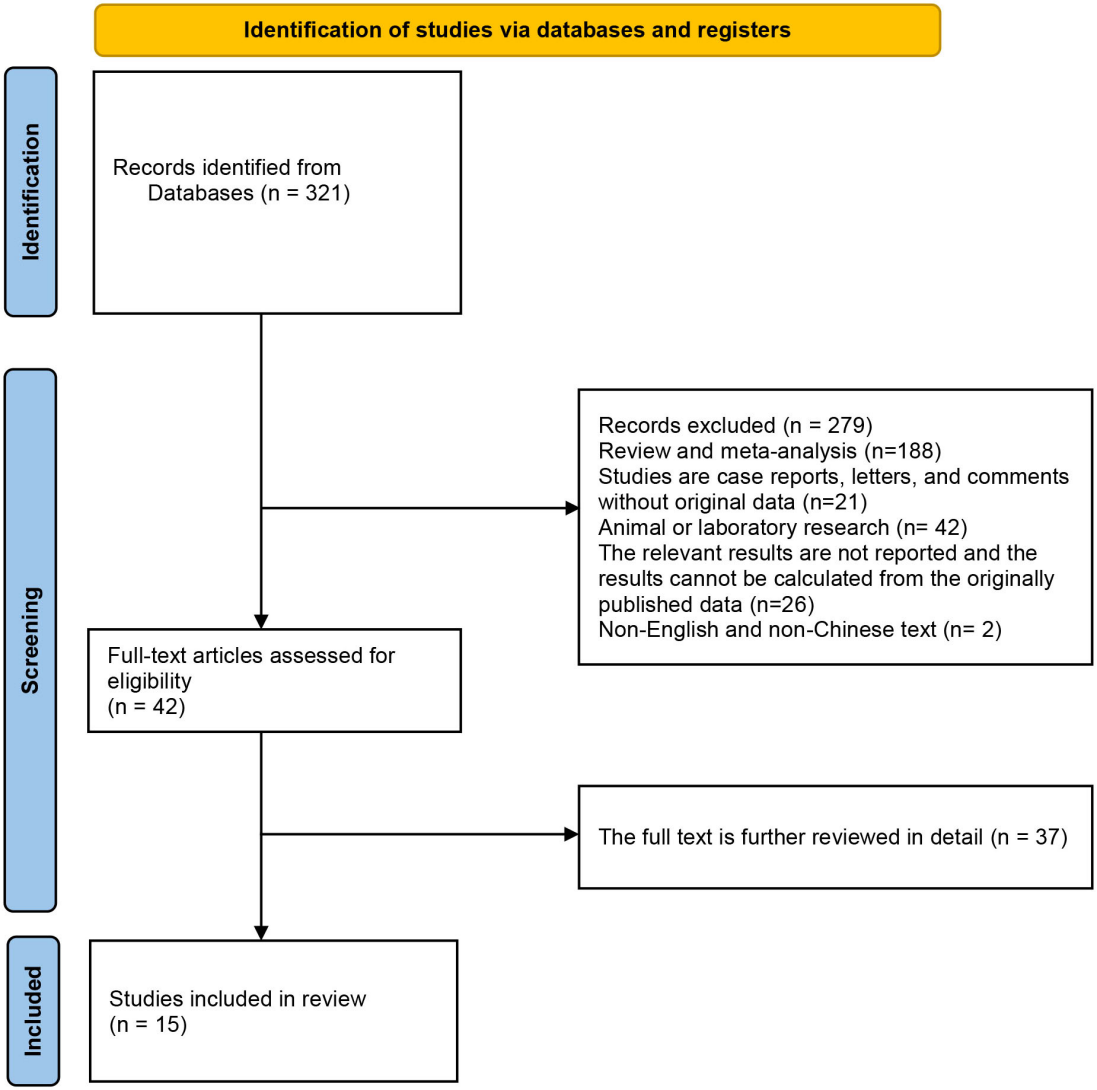
Identification of studies via databases and registers.

Initially, the database search yielded a total of 321 records eligible for inclusion. By looking at the titles or abstracts of all articles, 279 articles were excluded according to exclusion criteria (Exclusion: (1) Review and meta-analysis; (2) Studies are case reports, letters, and comments without original data; (3) Animal or laboratory research; (4) The relevant results are not reported and the results cannot be calculated from the originally published data; (5) Repeat studies based on the same database or patients. (6) Non-English or Chinese studies.). After further detailed review of the remaining 42 articles, 15 studies that met the inclusion criteria were finally included in this meta-analysis. The flow chart of research selection is shown in **Figure 1** (20–34).

### Study selection and characteristics

The baseline characteristics of the included studies are shown in **Table 1** (including tissue source, treatment received by patients, sample size, HER2 positive detection rate, detection methods and histopathological types). The included studies were published between 2006 and 2024, and each study included 50 to 306 patients. in addition, HER2 expression levels were measured in tumor tissues across all 15 studies by immunochemical staining (IHC), Fluorescence *in situ* hybridization, (FISH), or Next-generation sequencing (NGS). The definition of HER2 overexpression in the literature is ① IHC: HER2 (+++); FISH: HER2 amplification; ③NGS: HER2 gene positive (20–34).

**Table 1:** Main characteristics of the studies included in this meta-analysis.

Author	Year	Patient source	Tissues source	Treatment	case	HER2 (+)patients (%)	Method	HER2 Positive expression	Pathological differentiation
Yasumasa (20)	2006	Japan	Extrahepatic cholangiocarcinoma Gallbladder adenocarcinoma	Surgery	97	25.8	IHC	IHC(3+)	–
Kim (21)	2007	Korea	Extrahepatic cholangiocarcinoma	Surgery	55	29.1	IHC	IHC(3+)	Adenocarcinoma
Harder (22)	2009	Germany	Gallbladder cancer Intrahepatic cholangiocarcinoma Extrahepatic cholangiocarcinoma	Surgery	124	20.2	IHC	IHC(3+)	Adenocarcinoma
Yao (23)	2017	China	Gallbladder adenocarcinoma	Surgery	123	20.9	IHC、FISH	IHC(3+) FISH amplification: HER2 gene positive	Adenocarcinoma
Yang (24)	2018	China	Gallbladder carcinoma	Surgery	57	42.5	IHC	IHC(3+)	/
Albrecht (25)	2019	Germany	Gallbladder carcinoma	Surgery	186	5.4	IHC	IHC(3+)	Tubular adenocarcinoma Intraductal papillary neoplasm with an associated invasive carcinoma Intestinal type adenocarcinoma Mucinous carcinoma Adenosquamous carcinoma Clear cell carcinoma Signet ring cell carcinoma Sarcomatoid carcinoma
Zhan (26)	2019	China	Gallbladder adenocarcinoma	Surgery	50	14.0	IHC	IHC(3+)	–
Li (27)	2020	China	Gallbladder carcinoma	Surgery	60	20.0	IHC	IHC(3+)	Adenocarcinoma
Lee (28)	2020	Korea	extrahepatic cholangiocarcinoma	Surgery	230	5.7	IHC	IHC(3+)	Tubular adenocarcinoma Intraductal papillary neoplasm with an associated invasive carcinoma Intestinal type adenocarcinoma Mucinous carcinoma Adenosquamous carcinoma Clear cell carcinoma Signet ring cell carcinoma Sarcomatoid carcinoma
Vivaldi (29)	2020	Italy	intrahepatic cholangiocarcinoma、extrahepatic cholangiocarcinoma、 gallbladder carcinoma	Surgery	100	11.0	IHC、FISH	IHC(3+) FISH amplification: HER2 gene positive	–
Kim (30)	2022	Korea	Advanced biliary malignancy	Gemcitabine and CISPLATIN (GP)	121	14.9	NGS	NGS amplification: HER2 gene positive	–
Chen (31)	2022	China	Gallbladder carcinoma	Surgery	306	11.8	IHC、FISH	IHC(3+) FISH amplification: HER2 gene positive	adenocarcinoma adenosquamous carcinoma squamous cell carcinoma intraepithelial neoplasia of high grade tumor in situ mixed neuroendocrine/non-neuroendocrine neoplasm neuroendocrine neoplasm poor coesive carcinoma/signet ring cell carcinoma mucinous adenocarcinoma
Verma (32)	2023	India	advanced gallbladder carcinoma	Not receiving any systemic therapy (radiation, chemotherapy, or targeted therapy)	50	20.0	IHC、FISH	IHC(3+) FISH amplification: HER2 gene positive	Adenocarcinoma Mucinous adenocarcinoma Squamous cell carcinoma Small cell carcinoma Sarcomatoid carcinoma
Ma (33)	2023	China	Intrahepatic cholangiocarcinoma	Surgery	50	68.0	IHC、FISH	IHC(3+) FISH amplification: HER2 gene positive	–
Yeseul (34)	2024	Korea	Gallbladder cancer Perihilar cholangiocarcinoma Intrahepatic cholangiocarcinoma	Surgery	192	22.4	IHC	IHC(3+)	Adenocarcinoma Squamous/adenosquamous Sarcomatoid/undifferentiated

NGS, Next-generation sequencing.

IHC, Immunohistochemistry.

FISH, Fluorescence *in situ* hybridization.

### HER2 overexpression and clinicopathological features

**Figure 2:**
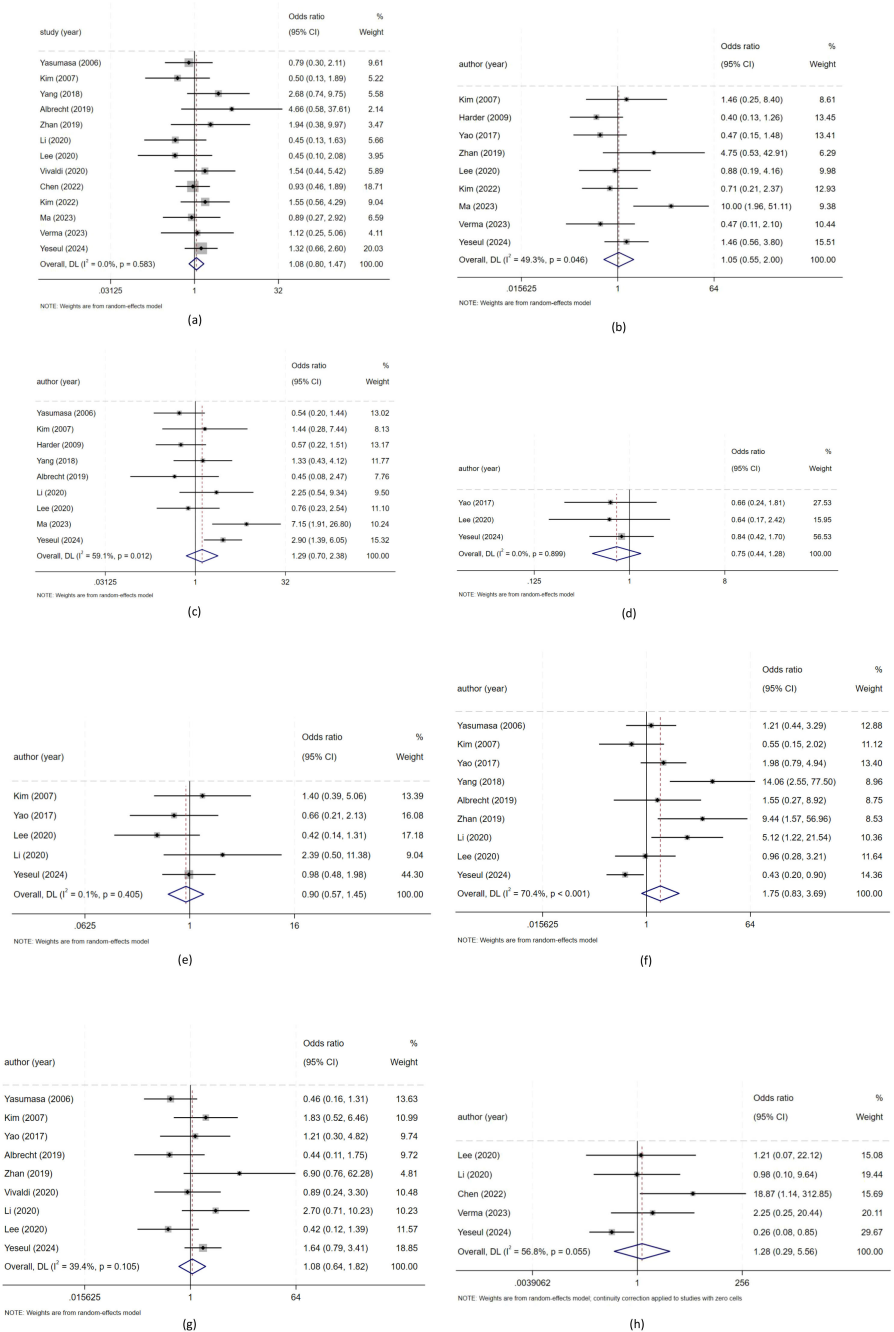
The relationship between positive/high expression of HER2 and clinical characteristics of patients withbiliary tract malignant tumors. **(a)** Meta-analysis of the association between HER2 overexpression and gender for biliary tract malignant tumors patients; **(b)** Meta analysis of the association between HER2 overexpression and tumor differentiation for biliary tract malignant tumors patients; **(c)** Meta analysis of the association between HER2 overexpression and tumor stage for biliary tract malignant tumors patients; **(d)** Meta analysis of the association between HER2 overexpression and vascular invasion for biliary tract malignant tumors patients; **(e)** Meta analysis of the association between HER2 overexpression and nerve invasion for biliary tract malignant tumors patients; **(f)** Meta analysis of the association between HER2 overexpression and lymph node metastasis for biliary tract malignant tumors patients; **(g)** Meta analysis of the association between HER2 overexpression and T stage for biliary tract malignant tumors patients; **(h)** Meta analysis of the association between HER2 overexpression and pathological type for biliary tract malignant tumors patients);.

In this study, we evaluated the relationship between positive/high expression of HER2 and clinicopathological features in patients with biliary tract carcinoma. We used a random effects model for meta-analysis. There was no significant statistical relationship between positive/high expression of HER2 and a series of clinical characteristics including gender, high, middle and low differentiation, tumor stage, vascular invasion, nerve invasion, Lymph node metastasis, T stage and pathological type of patients with biliary tract carcinoma. As shown in **Figure 2**. *(a: gender; b: high, middle and low differentiation; c: tumor stage; d: vascular invasion; e: nerve invasion; f: lymph node metastasis; g: T stage; h: pathological type of patients with biliary tract carcinoma)*, Heterogeneity: I² or i-squared; p<0.05 indicates statistical significance, otherwise no.

### HER2 overexpression and prognostic values

**Figure 3:**
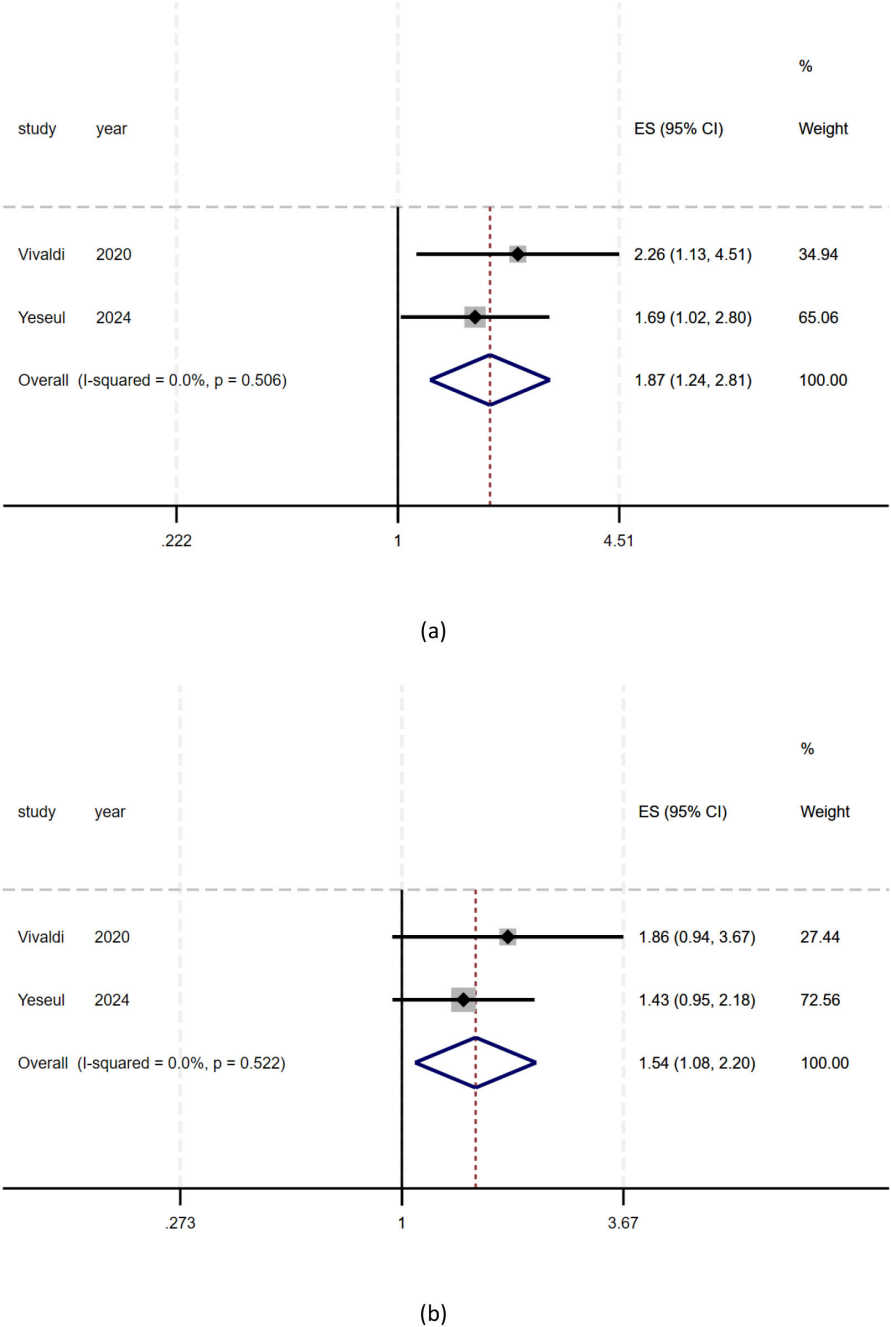
The relationship between positive/high expression of HER2 and prognosis of patients with biliary tract cancer. **(a)** Meta analysis of HER2 overexpression and DFS in patients with biliary tract cancer; **(b)** Meta analysis of HER2 overexpression and OS in patients with biliary tract cancer.).

As shown in **Figure 3**, there was a significant relationship between positive/high expression of HER2 and postoperative DFS (Disease-Free Survival) in patients with biliary tract carcinoma (ES = 1.87, 95% CI: 1.24-2.81, p = 0.003), the DFS of patients with positive/high expression of HER2 was 1.87 times that of patients with negative/low expression of HER2. There was a significant relationship between positive/high expression of HER2 and postoperative OS (Overall Survival) in patients with biliary tract carcinoma (ES = 1.54, 95% CI: 1.08-2.20, p = 0.017), the OS (Overall Survival) of patients with positive/high expression of HER2 was 1.54 times that of patients with negative/low expression of HER2. As shown in **Figure 3**. (ES: Effect Sizes). Heterogeneity: I² or i-squared; p<0.05 indicates statistical significance, otherwise no.

### Quality assessment

The Newcastle Ottawa Quality Assessment Scale assessed research quality between 6 and 7. The NOS scores of all enrolled studies were >5, indicating a relatively high quality of research methods (**Table 2**) (35).

**Table 2:** The Newcastle-Ottawa scale (NOS) quality assessment of the enrolled studies.

References	Selection				Comparability		Outcome			Total
	Representativeness of the exposed cohort	Selection of the non-exposed cohort	Ascertainment of exposure	Demonstration that outcome of interest was not present at start of study	Comparability of cohorts on the basis of the design or analysis	Comparability of cohorts on the basis of the measurement	Assessment of outcome	Was follow-up long enough for outcomes to occur	Adequacy of follow up of cohorts	
Yasumasa, 2006 (20)	0	1	1	0	1	1	1	0	1	6
Kim, 2007 (21)	0	1	1	0	1	1	1	1	1	7
Harder, 2009 (22)	0	1	1	0	1	1	1	0	1	6
Yao, 2017 (23)	0	1	1	0	1	1	1	1	1	7
Yang, 2018 (24)	0	1	1	0	1	1	1	1	1	7
Albrecht, 2019 (25)	0	1	1	0	1	1	1	0	1	6
Zhan, 2019 (26)	0	1	1	0	1	1	1	1	1	7
Li, 2020 (27)	0	1	1	0	1	1	1	1	1	7
Lee, 2020 (28)	0	1	1	0	1	1	1	1	1	7
Vivaldi, 2020 (29)	0	1	1	0	1	1	1	1	1	7
Kim, 2022 (30)	0	1	1	0	1	1	1	1	1	7
Chen, 2022 (31)	0	1	1	0	1	1	1	0	1	6
Verma,2023 (32)	0	1	1	0	1	1	1	0	1	6
Ma, 2023 (33)	0	1	1	0	1	1	1	1	1	7
Yeseul, 2024 (34)	1	1	1	0	1	1	1	1	1	7

### Publication bias

**Figure 4:**
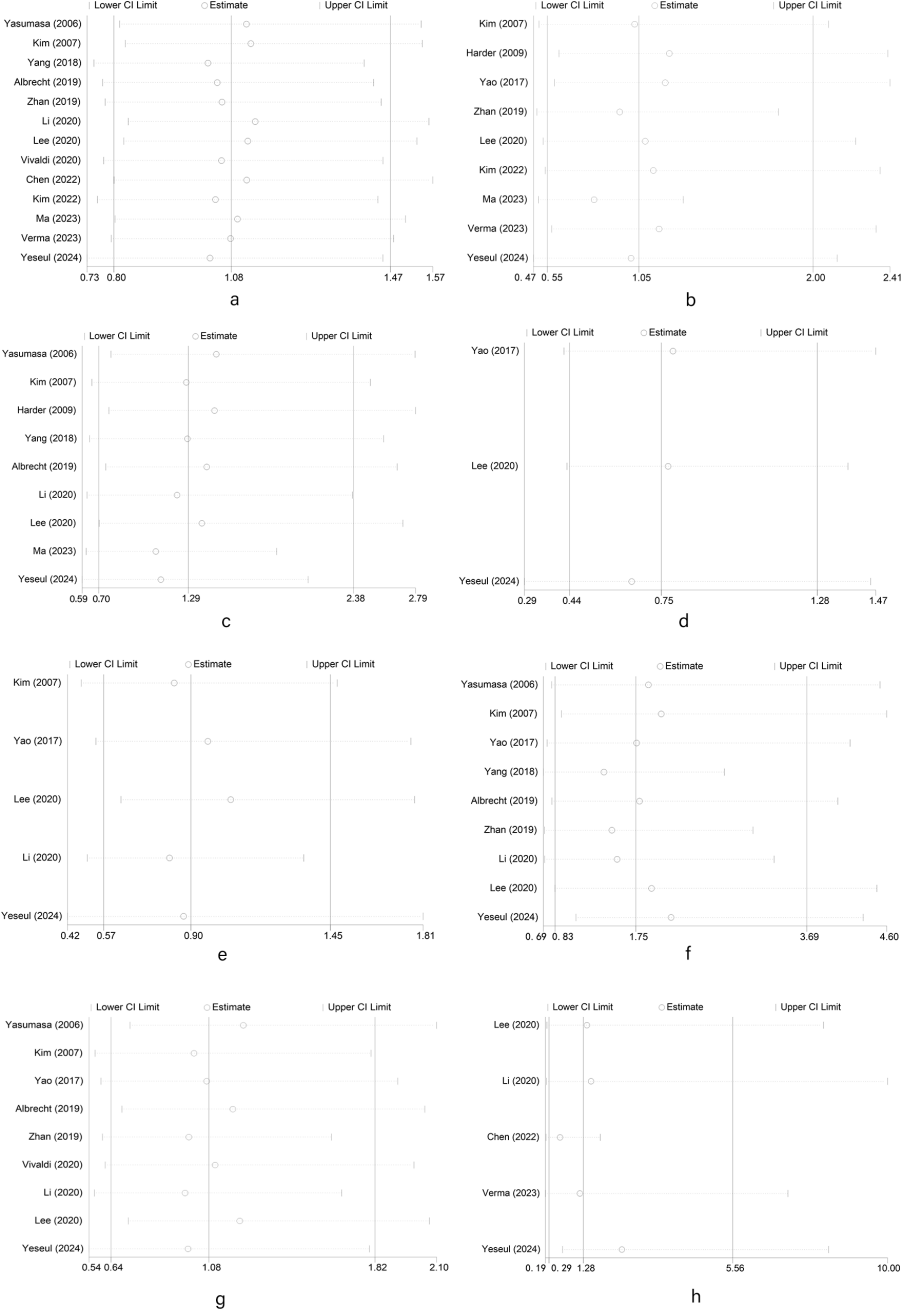
Random-effects model in sensitivity analysis for publication bias. **(a)** Sensitivity analysis of meta analysis of HER2 overexpression and gender in patients with biliary tract malignant tumors; **(b)** Sensitivity analysis of meta analysis of HER2 overexpression and tumor differentiation in patients with biliary tract malignant tumors; **(c)** Sensitivity analysis of meta analysis of HER2 overexpression and tumor stage in patients with biliary tract malignant tumors; **(d)** Sensitivity analysis of meta analysis of HER2 overexpression and vascular invasion in patients with biliary tract malignant tumors; **(e)** Sensitivity analysis of meta analysis of HER2 overexpression and nerve invasion in patients with biliary tract malignant tumors; **(f)** Sensitivity analysis of meta analysis of HER2 overexpression and lymph node metastasis in patients with biliary tract malignant tumors; **(g)** Sensitivity analysis of meta analysis of HER2 overexpression and T stage in patients with biliary tract malignant tumors; **(h)** Sensitivity analysis of meta analysis of HER2 overexpression and pathological type in patients with biliary tract malignant tumors.).

We used a random-effects model in sensitivity analysis (as shown in **Figure 4**) *(a: gender; b: high, middle and low differentiation; c: tumor stage; d: vascular invasion; e: nerve invasion; f: lymph node metastasis; g: T stage; h: pathological type of patients with biliary tract carcinoma)*, deleting each study in each round, to further determine the validity of HER2’s prognostic effects. As shown in **Figure 4**, after deleting any of the studies, the results of merging OR remain unchanged. These results indicate that it is robust that positive/high expression of HER2 is not significantly correlated with a series of clinical features such as gender, high, middle and low differentiation, tumor stage, vascular invasion, nerve invasion, T stage, and pathological type of patients with biliary tract carcinoma. Due to the limited number of studies examining OS and DFS, no sensitivity analysis was performed. All outcome indicators in this study included fewer than 10 studies, so publication bias analysis is meaningless (36).

## Discussion

This study revealed the association between biliary tract carcinoma and positive/high expression of HER2 and clinicopathology and prognosis. Our meta-analysis suggested that, compared with low HER2 overexpression, there was no significant correlation between HER2 overexpression and pathological features in biliary tract carcinoma. The positive/high expression of HER2 in biliary tract carcinoma was significantly correlated with the prolongation of OS and DFS. HER2 The positive/high expression of HER2 in biliary malignancies was significantly correlated with the shortening of OS and DFS. Patients with HER2 positive/highly expressed biliary tract carcinoma had shorter OS and DFS and worse prognosis.

As we all know, HER2 is a family of epidermal growth factor receptors A member of receptor (EGFR) and the other three members of the receptor family (HER1, HER3 and HER4) can activate the downstream tyrosine kinase signaling cascade after dimerization with a variety of peptide ligands, and initiate the MAPK pathway, AKT pathway and p70S6K/p85S6K pathway, etc. The signaling range includes cell division and migration Metastasis (all associated with tumorigenesis) to adhesion, differentiation, and apoptosis (37). HER2 has no ligand of its own, but the heterodimer it binds to the other three EGF receptors is the most efficient complex in the ErbB signaling network. Overexpression of HER2 has been found in a variety of solid tumors, such as breast cancer, stomach cancer, cholangiocarcinoma, colorectal cancer, non-small cell lung cancer, endometrial cancer, and bladder cancer, with positive rates ranging from more than 50% in endometrial cancer to 2% in non-small cell lung cancer, but there is significant inter-tumor heterogeneity in HER2 overexpression patterns (38, 39). In the breast cancers with the most mature and accurate HER2 research and clinical transformation, 20% to 30% of breast cancers have HER2 overexpression, and it is associated with more aggressive tumor, higher recurrence rate and shorter survival time. Trastuzumab, a monoclonal antibody targeting HER2, has become the standard treatment for patients with HER2 overexpression breast cancer (40, 41). Favorable clinical results of anti-HER2 antibodies in breast cancer have led to analysis of HER2 overexpression in other solid tumors (42). HER2 overexpression have also been detected in ovarian, lung, stomach, and colon cancers (43).

For biliary tract carcinoma biliary tract carcinoma biliary tract carcinoma, a meta-analysis showed that Asian patients had a higher rate of HER2 overexpression than Western patients (28.4% vs 19.7%) (44). In a Japanese study, the positive rate of HER2 varies among different subtypes of cholangiocarcinoma: 3.7% for intrahepatic cholangiocarcinoma, 3% for proximal extrahepatic cholangiocarcinoma, 18.5% for distal extrahepatic cholangiocarcinoma, 31.3% for gallbladder carcinoma, and 16.4% for ampulla carcinoma (45). However, regarding the relationship between HER2 and prognosis of biliary tract carcinoma, there are conflicting results. Several studies have shown that reported HER2 overexpression is associated with late stage, reduced survival, or both (20–34).

Both HER2 and EGFR belong to the ErbB receptor tyrosine kinase family. Overexpression of HER2 can lead to receptor dimerization (autologous or allogeneic). After receptor dimerization, autophosphorylation occurs in the cytoplasmic segment of ErbBs, which then transmits signals downstream to activate the ERK signal transduction pathway and the PI3K/AKT signal transduction pathway (46). At the same time, HER2 can also block the internalization and degradation of EGFR, resulting in increased EGFR expression level (45). By activating downstream oncogenic signaling networks such as PI3K/AKT, MAPK/ERK, and STAT3, HER2 overexpression remodels the tumor microenvironment and induces epithelial-mesenchymal transition (EMT), thereby promoting tumor invasion, metastasis, and treatment resistance. Specifically, HER2 overexpression activates the PI3K/AKT signaling pathway through homodimerization or heterodimerization. PI3K catalyzes the production of PIP3, recruiting AKT to the cell membrane and phosphorylating its Thr308 and Ser473 residues. This process activates downstream effector molecules, including mTOR and GSK-3β, inhibits pro-apoptotic protein BAD, and promotes glycolysis. Under hypoxic conditions, AKT stabilizes HIF-1α, enhancing tumor cell survival while inducing VEGF secretion and promoting angiogenesis. Furthermore, tumor cells activated by the PI3K/AKT pathway secrete IL-6 and TGF-β, recruiting cancer-associated fibroblasts (CAFs) and stimulating their collagen secretion, thereby forming a pro-fibrotic extracellular matrix. Additionally, this pathway upregulates PD-L1 expression, establishing an immunosuppressive microenvironment via inhibition of T cell function. HER2 also activates the RAS-RAF-MEK-ERK pathway, which enhances Cyclin D1 expression and suppresses p27. ERK directly promotes Cyclin D1 transcription by activating transcription factors such as AP-1, forming complexes with CDK4/6 to drive the cell cycle into the S phase. Simultaneously, ERK accelerates p27 proteasomal degradation through phosphorylation, inhibits its transcription, and releases the suppression of CDK2 by p27, further facilitating G1/S phase transition. The deletion of p27 leads to continuous activation of CDK2, jointly propelling rapid cell cycle progression and promoting tumor cell proliferation and survival. Overall, HER2 overexpression reshapes the immune microenvironment and drives EMT through a multidimensional signaling network (47–49). Therefore, overexpression of HER2 can over activate downstream signal transduction pathways, thus promoting the occurrence and development of cancer.

No meta-analysis on the correlation between HER2 overexpression and clinicopathologic features of biliary malignancies could be found in the database used in this search. This is the first meta-analysis to evaluate the correlation between HER2 overexpression and clinicopathologic features of biliary tract carcinoma. It has the characteristics of large sample size and relatively small heterogeneity, which makes the results obtained by statistical analysis more accurate and convincing, and can fully prove the correlation between HER2 overexpression level and clinicopathological features of biliary tract carcinoma. Among them, there was no significant correlation between the high expression of HER2 gene and the degree of differentiation, TNM stage, lymph node metastasis, vascular invasion, nerve invasion, pathological type, T stage and gender wind grouping. Further sensitivity analysis using one-by-one elimination method proved that the results were robust, indicating that the conclusions reached in this study were reliable.

In the studies included, various detection platforms utilized distinct immunohistochemical techniques for protein expression analysis. These differences encompassed antibody types and concentrations, threshold definitions, standardization of detection procedures, quality control product consistency, and subjective scoring biases, all of which are critical for HER2 evaluation. In this paper, HER2 expression was predominantly detected via immunohistochemistry (IHC). When HER2 IHC (2+) results were obtained, fluorescence *in situ* hybridization (FISH) interpretation was performed as a supplementary measure to IHC. However, FISH detection lacked sufficient cross-validation with IHC, limiting its reliability. Since the studies included did not comprehensively compare the consistency between IHC and FISH in determining HER2 positivity/negativity, the influence of accidental consistency could not be corrected, and the reliability of the detection methods remained unexplored. Due to insufficient data and lack of cross-validation among the included studies, stratified subgroup analyses based on detection methods could not be conducted, potentially reducing statistical efficiency. The inability to exclude the impact of methodological differences through subgroup analysis may enlarge the confidence interval of combined effect sizes (e.g., OR, HR) and increase heterogeneity test results, thereby diminishing the credibility of the conclusions. These limitations significantly increased outcome heterogeneity, reduced the reference value for treatment decisions, and decreased cross-study comparability. We recommend that future studies clearly specify antibody clone numbers, detection platforms, and quality control processes (e.g., the use of standardized products), facilitating subsequent secondary analyses. Additionally, cross-validation data for IHC and FISH should be supplemented for key studies or next-generation sequencing (NGS) technology employed to assist in verifying HER2 status. Furthermore, the studied populations may not accurately represent global HER2 expression due to a lack of rich data, complicating the establishment of a standard measurement for HER2 status.All studies included in this analysis were retrospective in design, inherently prone to flaws that may weaken the strength of evidence. Retrospective studies are susceptible to selection bias (e.g., institutional admission criteria disparities) and information bias (e.g., incomplete medical records), particularly concerning HER2 detection procedures. Significant heterogeneity exists in IHC interpretation criteria and FISH amplification thresholds across studies, potentially leading to systematic amplification of positive rate differences. Although numerical differences in HER2 status were observed among the included studies, statistical verification was unfeasible due to uneven geographical distribution in the original data (e.g., absence of data from Africa and South America) and the lack of stratified clinical features in most studies. Regional differences may stem from etiological backgrounds: in East Asia, biliary calculi and chronic biliary inflammation are major causes of cholangiocarcinoma (6), whereas European cases are often associated with bile duct malformations or metabolic syndrome, suggesting differing oncogenic mechanisms (50). Moreover, ethnic genetic heterogeneity may influence HER2 protein stability, yet no existing studies incorporated genotype data (51). Future research urgently requires multinational prospective cohort studies employing standardized testing procedures (e.g., synchronous IHC/FISH testing and NGS validation) and systematically collecting regional environmental exposure, host genetic variation, and molecular typing data to more accurately elucidate the biological heterogeneity and clinical significance of HER2 in biliary tract carcinoma.The efficacy of HER2-targeted drugs in gastric cancer and breast cancer patients with HER2 gene expression has been well-documented. As precision medicine continues to gain acceptance, there is growing interest in exploring the potential application of anti-HER2 therapies in other cancers, including biliary tract carcinoma (52). Currently, advanced biliary tract carcinoma treatment involves four main categories of anti-HER2 drugs: monoclonal antibodies, small-molecule HER1/2 tyrosine kinase inhibitors, antibody-drug conjugates, and dual-clonal antibodies (52). The NCCN guidelines recommend three regimens for HER2 overexpression BTC patients: trastuzumab (IHC3+), trastuzumab + pertuzumab, and tucatinib + trastuzumab. These recommendations are supported by studies such as DESTINY-PanTumor02, MyPathway, and SGNTUC-019, which reported overall objective response rates (ORRs) ranging from 23% to 46.7%. Among these, anti-HER2 monoclonal antibodies, exemplified by trastuzumab and pertuzumab, inhibit downstream HER2 signaling pathways by binding to specific HER2 domains, thereby exerting anti-tumor effects. However, HER2-targeted small molecule agents like lapatinib, varlitinib, and neratinib, either alone or in combination with capecitabine, have not demonstrated significant clinical benefits for biliary tract carcinoma, necessitating further investigation into more effective combination therapies (53, 54). Notably, the combination of tucatinib and trastuzumab achieved a confirmed ORR of up to 46.7% in HER2 overexpression biliary tract carcinoma (SGNTUC-019), highlighting its potential for improving therapeutic outcomes (55).

In November 2024, the U.S. FDA granted accelerated approval to zanidatamab, the world’s first HER2 dual-epitope dual antibody, for treating previously treated, unresectable, or metastatic HER2 overexpression (IHC 3+) biliary tract cancer in adults. This approval was based on robust data from the HERIZON-BTC-01 clinical trial, which enrolled 87 patients with unresectable, locally advanced, or metastatic HER2 overexpression BTC. With a median follow-up of 12.4 months, the trial results demonstrated an objective response rate (ORR) of 41.3% (95% CI 30.4-52.8), a median duration of response (DoR) of 12.9 months, and a median progression-free survival of 5.5 months (56). These outcome data indicate that HER2 amplification may serve as a potentially treatment-relevant target for patients with advanced biliary tract cancer (BTC), given that anti-HER2 therapy confers a survival benefit in this patient population.

The analysis of HER2 overexpression level and OS and DFS in biliary tract carcinoma showed that the OS and DFS in patients with low HER2 gene expression were 1.54 times and 1.87 times higher than those in patients with HER2 gene amplification/overexpression, respectively. HER2 gene patients have a high recurrence rate and a poor prognosis. Patients with low HER2 gene expression may have a good prognosis and a low recurrence rate. This also tip HER2 amplification is BTC a biological marker of poor prognosis. This clinical result is in line with the molecular biological manifestations of HER2 in biliary tract carcinoma. A retrospective study conducted in Italy on 100 patients with biliary tract carcinoma resection found significant differences in the mOS of various types of BTC, with ampulla cancer not reaching mOS. The mOS of intrahepatic cholangiocarcinoma was 55.3 months. The median mOS of extrahepatic cholangiocarcinoma was 34.7 months. The mOS for gallbladder cancer was 18.1 months, and these differences suggest that the primary site of BTC may be an important factor in determining patient prognosis (29). Positive/high expression of HER2 is not significantly correlated with a series of clinical features such as gender, high, middle and low differentiation, tumor stage, vascular invasion, nerve invasion, T stage, and pathological type of patients with biliary tract carcinoma. But results from a Korean study of cholangiocarcinoma patients after surgery suggest: The expression rate of HER2 protein appears to be lower in extrahepatic cholangiocarcinoma compared to the results of studies in intrahepatic cholangiocarcinoma. And overexpression of HER2 protein induced by HER2 gene amplification may constitute an independent prognostic factor in patients with lymph node metastasis, as growth factors released during postoperative wound healing have been shown to preferentially stimulate the growth of HER2/newly positive tumors. These growth factors are more likely to have a stimulating effect in patients with diffuse micrometastases (lymph node positive patients) with HER2 amplified tumors, which may also explain the effect of HER2 on prognosis depending on lymph node status (21). Relevant studies have reported that the HER2 pathway may play a role in the development and growth of BILIARY TRACT CARCINOMA, and activating the overexpression of HER2 can lead to the development of biliary tract carcinoma (57). Inflammation and calculi in the biliary system can activate the EGFR/HER2 pathway, which in turn can lead to the proliferation and invasion of malignant biliary tumors, which may further promote the expression of this pathway and form a vicious cycle (58). This suggests that the expression of HER2 is closely related to the proliferation, development, metastasis, invasiveness and drug resistance of biliary malignant tumor cells, which is of great significance in the evaluation and treatment of biliary malignant tumor. However, since only two studies have been retrieved with reports of DFS and OS, more studies on HER2 gene expression and DFS and OS in biliary tract carcinoma need to be carried out worldwide to better guide prognosis.

## Conclusion

This meta-analysis showed that high expression of HER2 gene was not correlated with clinicopathological parameters such as differentiation degree, TNM stage, lymph node metastasis, vascular invasion, nerve invasion, pathological type, T stage, and gender of biliary tract carcinoma. HER2 overexpression is a negative prognostic factor in biliary tract carcinoma patients. In the future, well-designed clinical studies of large cases of biliary tract carcinoma should be carried out to verify the relationship between HER2 overexpression and pathological features and prognosis of biliary tract carcinoma patients.

## Data Availability

The original contributions presented in the study are included in the article/supplementary material. Further inquiries can be directed to the corresponding author/s.
